# A brief history of galectin evolution

**DOI:** 10.3389/fimmu.2023.1147356

**Published:** 2023-06-29

**Authors:** Juliane Günther, Sebastian Peter Galuska

**Affiliations:** Glycobiology Unit, Institute of Reproductive Biology, Research Institute for Farm Animal Biology (FBN), Dummerstorf, Germany

**Keywords:** galectin, lectins, glycans, evolution, vertebrate, galactose binding lectin, sialic acid binding, galectin-8

## Abstract

Galectins are a family of carbohydrate-binding proteins found in vertebrates in great abundance and diversity in terms of both structure and ligand-binding properties as well as physiological function. Proteins with clear relationships to vertebrate galectins are already found in primitive Bilateria. The increasing amount of accessible well-annotated bilaterian genomes has allowed us to reveal, through synteny analyses, a new hypothesis about the phylogenetic history of the galectin family in this animal group. Thus, we can trace the genomic localization of the putative ancestral Bilateria galectin back to the scallops as a still very primitive slow-evolving bilaterian lineage. Intriguingly, our analyses show that the primordial galectin of the Deuterostomata most likely exhibited galectin-8-like characteristics. This basal standing galectin is characterized by a tandem-repeat type with two carbohydrate recognition domains as well as by a sialic acid binding property of the N-terminal domain, which is typical for galectin-8. With the help of synteny, the amplification of this potential primordial galectin to the broad galectin cosmos of modern jawed vertebrates can be reconstructed. Therefore, it is possible to distinguish between the paralogs resulting from small-scale duplication and the ohnologues generated by whole-genome duplication. Our findings support a substantially new hypothesis about the origin of the various members of the galectin family in vertebrates. This allows us to reveal new theories on the kinship relationships of the galectins of Gnatostomata. In addition, we focus for the first time on the galectines of the Cyclostomata, which as a sister group of jawed vertebrates providing important insights into the evolutionary history of the entire subphylum. Our studies also highlight a previously neglected member of the galectin family, galectin-related protein 2. This protein appears to be a widespread ohnologue of the original tandem-repeat ancestor within Gnathostomata that has not been the focus of galectin research due to its nonclassical galactose binding sequence motif and the fact that it was lost during mammalian evolution.

## 1 Introduction

Galectins are small glycan-binding proteins that originally received their name because of their galactose-binding property. These lectins are widespread in the animal kingdom. The Porifera already possess galectin-like proteins (1). However, the formation of a multifaceted galectin family is found only in Bilateria. These proteins share a complex secondary structure composed of β-sheets (named S1-S6 and F1-F5), which form the carbohydrate recognition domain (CDR) (2, 3). Only the S-strands and the loop regions connecting them and form together the glycan binding groove. The galectin cosmos of Gnathostomata is particularly complex. Based on structural features, galectins are assigned to three subgroups. The prototypical galectins possess only a single CRD. The same is true for the chimeric galectins. However, these proteins additionally have a longer N-terminal binding domain, which enables them to form pentamers. Tandem-repeat galectins, on the other hand, have two CRDs connected by a linker region that can vary in size. In addition, galectin-related proteins (GRPs) structurally correspond to prototypical galectins. However, minor amino acid changes in the specific binding motifs resulted in a loss of their galactose-binding capacity (4). Based on the overall similarity of the CRD at the protein and gene structure level, it can be assumed that the GRPs can be readily assigned to the galectin family.

Each galectin or, better, each galectin CRD of Gnathostomata has its own carbohydrate-binding preference. For instance, the N-terminal CRD of galectin-8 preferentially binds sulfated and sialylated carbohydrates (5). The C-terminal domain of this galectin, on the other hand, has a preference for histo-blood group antigen-like and poly-N-acetyllactosamine glycans (6). In addition, many galectins have been shown to interact with a protein-binding partner. Prototypic galectins, for example, form homo or heterodimers with each other but can also form dimers with chemokines (7). Another well-known example is the binding of galectin-8 with the autophagy receptor NDP52. This interaction is a crucial step in galectin-mediated selective autophagocytosis after vesicle damage and critical in defense against intracellular pathogens (8, 9). Galectins can serve diverse functions both intracellularly and extracellularly (10). The role of galectins in the immune system is particularly multifaceted, ranging from pattern-recognition receptor function in pathogen recognition (11) to regulatory functions on specific immune cells (10). Several Gnatostomata galectins (including galectin-1, -3, and -9) have important roles in the regulation of the adaptive immune response. In particular, their relevance in T-cell activation and B-cell differentiation is noteworthy (12). Thus, a correlation between the expanding galectin family and the increased complexity of the immune system during vertebrate evolution might be speculated. The redundancy that occurs after whole genome duplication (WGD) usually results subsequently in massive gene loss (13, 14). The galectin ohnologues ubiquitously retained in the vertebrate genomes and diversified their function. This reflects their importance, probably in particular for the regulation of the immune system.

The various members of the galectin family have been well studied in mammals, especially in humans and mice, as the most prominent model animals. In addition, there are some studies on individual galectins, especially of Tetrapoda [e.g. chicken (15, 16) and Teleostei (17)].

It has long been known that the nomenclature of galectins based on structural similarities does not provide any information about their evolutionary relationship (18). Moreover, the previous hypothesis on the evolution of vertebrate galectins is largely based on the findings of Houzelstein et al. from 2004 (18). It was assumed [reviewed in (19)] that the last common ancestor of Protostomia and Deuterostomia possessed an ancestral galectin with a CRD that corresponds in exon structure to the N-terminal CRD (referred hereafter as CRD1) of modern Gnatostomata tandem-repeat galectins. In chordates, the C-terminal CRD (hereafter CRD2) with its typical exon structure, characterized by a shorter middle exon, is supposed to have evolved by tandem duplication. Thus, the protovertebrate ancestors owned a tandem-repeat galectin with two different CRDs (CRD1 and CRD2), the exon structure of which corresponds to that of modern tandem-repeat Gnatostomata galectins. In the course of the two rounds of WGD that occurred during Gnatostomata evolution, one tandem-repeat galectin became four. So that according to the current hypothesis the tandem-repeat galectins of the jawed vertebrates namely 4, 8, 9 and 12 represent ohnologues. In vertebrates small-scale gene duplication is supposed to have resulted in the formation of the mono-CRD galectins. Another hypothesis suggests that the mono-CRD galectins-1 and -2 are evolved from a prevertebrate mono-CRD galectin (20). The increasing quantity and quality of genomic data now makes it possible to look deeper into the phylogeny and evolution of galectins. These findings may improve our understanding of functional relationships in this protein family. To correctly identify the major galectins of Gnathostomata, we first looked at the genes surrounding individual galectins to identify overlapping syntenies. To understand how this variety of Gnathostomata galectins evolved during the evolution of Bilateria and where the original galectin locus is located, we used microsynteny studies as well as recent results on the reconstruction of the proto-vertebrate genome. Analyses of gene or protein structure also provide insights into the phylogenetic history of galectins. Finally, based on all these analyses, we can generate a model that explains the diversification of the various galectins of modern jawed vertebrates. To our knowledge, these findings allow us to propose the first substantial new hypothesis on the evolution of vertebrate galectins since 2004. This might help future research to develop new theories about the functional relationships of galectins based on their evolutionary context.

## 2 Material and methods

### 2.1 Sequences and genomic organization

Sequence information regarding the specific galectins and their syntenic genes, their genomic organization as well as chromosomal localization, and exon−intron structure was obtained from the NCBI gene and genome data viewer databases (https://www.ncbi.nlm.nih.gov/gene/ and https://www.ncbi.nlm.nih.gov/genome/gdv/). To cover the diverse Gnatostomata taxa, we manually screened the NCBI databases for the genomes of at least two representatives of each of the Chondrichthyes, Holostei (Actinopterygii without 3rd WGD, only *Lepisosteus oculatus* analyzed), Teleostei, Coelacanthidae (only *Latimeria chalumnae*), Dipnomorpha (only *Protopterus annectens*), Amphibia, Lepidosauria, Testudines, Aves, Monotremata, Metatheria, and Eutheria. We considered species whose genomes have a good assembly level. Exceptions are species that represent key stages of evolution such as *L. chalumnae* or *Callorhinchus milii*. These were used for the analyses despite the relatively low level of annotation.

For the macrosynteny analysis, the publication of Nakatani et al. (21) was used. By manual screening of the galectin localizations in the human genome, their positions could be assigned to the chromosomal regions derived from the respective scallop chromosomes.

### 2.2 Sequence alignments

Protein sequence alignments were performed using the Tcoffee algorithm implemented in Jalview version 2.11.2.5 (22).

### 2.3 Sequence-based phylogenetic analyses

Phylogenetic analyses were performed based on sequences of 222 galectin proteins (**Supplementary Table 1**). Amino acid sequences of CRD1 and CRD2 were aligned with Tcoffee algorithm. Maximum Likelihood phylogenetic tree with 1000 bootstrap replicates were generated using MEGA X (23) and edited by the iTOL tool (interactive tree of life) (24).

### 2.4 Protein structure analysis and molecular modeling

The structures of LGALS8-like proteins from *Petromyzon marinus* and *Lytechinus variegatus* were predicted using the intensive mode of the Phyre2 tool (25). The structure of human LGALS8 was obtained from the AlphaFold database (AF-Q96DT0-F1) (26).

Computation of the electrostatic potential using Adaptive Poisson Boltzmann Solver (APBS) and mapping to the molecular surface were performed using Phyton Molecule Viewer version 1.5.7 (https://ccsb.scripps.edu/mgltools/) (27–29).

## 3 Results and discussion

### 3.1 Genomic organization of Gnathostomata galectins

The increasing number of sequenced and well-annotated genomes allowed us to study the genomic distribution of galectins within Gnathostomata. Syntenic analyses helped to address identities of the galectin homologs up to the Chondrichthyes. We have restricted our analysis to the galectin-encoding genes (LGALS) that are present in a majority of the Gnathostomata classes, thus forming the backbone of the galectin family. This excludes, for example, LGALS10, which is found only in primates, or the large number of galectins similar to placenta-galectin, which are found almost exclusively in the Simiiformes. These are located in close proximity to LGALS4, just like LGALS7, which is present in many Amniota. **Table 1** shows the genes colocalized with the respective galectin in close proximity. A well-conserved synteny of galectins exists throughout jawed vertebrates. One exception is LGALS8, which exists in a different genomic context in Actinopterygii compared with Chondrichthyes and Sarcopterygii (30). Furthermore, in Chondrichthyes, we find only bloc1s3 in close proximity to galectin-4. Ryr1, actn4, ech1, and hnrnpl are located on the same chromosome, but they are separated by several million nucleotides. Moreover, LGALS12 is only found in Dipnotetrapodomorpha. The GRP LGALSL2, also called LGALSLA, exists in all Gnathostomata, but it has been lost in Mammalia.

**Table 1:** Syntenic genes of Gnathostomata galectins.

LGALS1 LGALS2	LGALS1B	LGALS3	LGALS4	LGALS8	LGALS8	LGALS9	LGALS12	LGALSL	LGALSL2	GRIFIN
			LGALS7, 13, 16, 14, 10*	Chondrichthyes & Sarcopterygii	Actinopterygii					
elfn2	shroom3	socs4	bloc1s3	ryr2	tmem242	nf1	hnrnpul2	ugp2	vti1b	chst12
gga1	septin11	fbxo34	hnrnpl	mtr	rrm2	wsb1	chrm1	vps54	zfyve26	lnfg
tmem184b	sqwahb	atg14	ech1	actn2	klf11	ksr1	naa40	peli1	plek2	eif3b
cacna1i	ccni	ktn1	actn4	heatr1	cebpz**	nos2	frmd8			brat1
cacng2		peli2	ryr1	cebpz**		lyrm9	pc			
						nlk				

* tandem dublication of LGALS4 N-terminal CRD.

** in the wider vicinity.

LGALS1B, which is structurally closely related to LGALS1, is unique to Sauropsida (reptiles and birds). In these animals, it is located on a different chromosome than LGALS1. In Tetrapoda, LGALS1 and LGALS2 are in close genomic proximity to each other. Although galectins are present in the genomic context of LGALS1 or LGALS2 in all gnathostomes, only Telostomi has a galectin, with an amino acid sequence typical of LGALS1 in the glycan-binding groove that spans β-strands S4 and S5 and loop L4 between them (31). A galectin similar in this important glycan-binding region to LGALS2 of tetrapods is already found in west African lungfish, but on Chr 1 part0 (NC_056725.1), the LGALS1-like LOC122794646 and the LGALS2-like LOC122801768 are approximately 700 mio nucleotides apart. In most of the Gnastostomata, LGALSL with LGALS8 and LGALSL2 with LGALS3 are located on one chromosome, although not in close proximity. In Mammalia, LGALS3 and LGALSL are found on different chromosomes, while LGALSL2 appears to be lost.

### 3.2 Origin of vertebrate galectins since the emergence of bilateria

**Figure 1:**
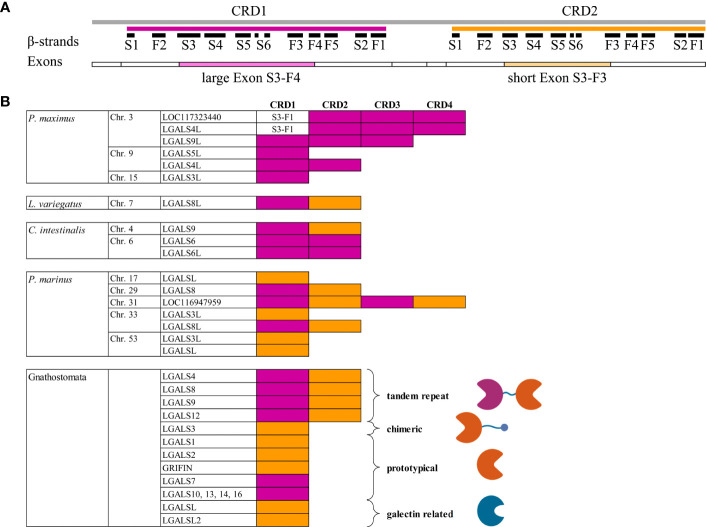
Comparison of Galectin Gene Organization. **(A)** Graphical illustration of the protein and gene structure of human galectin-8. Positions of the six S- and five F-strands of the first (magenta) and second (orange) CRD are shown as black bars. The distribution of the individual coding exons is represented as boxes below. The long and short middle exons that distinguish between the two CRDs and encode crucial ligand binding motifs are highlighted in color. **(B)** Presence of the different CRD subtypes within the galectins of *P. maximus*, *L. variegatus*, *C intestinalis*, *P. marinus* and the conserved Gnathosthomata proteins. The CRDs of the respective galectins are colored magenta or yellow, depending on whether they are encoded by the large middle exon or the small exon, respectively. For the Gnathostomata galectins, the schematic structure of the individual galectin subtypes is shown to the right of the CRDs. Created with BioRender.com.

Scallops are genomically well annotated and exhibit relatively slow genome evolution, making them good model organisms for studying the evolution of bilaterians (32). For this reason, we examined the genomic distribution and structure of galectins from the bivalve *Pecten maximus* to gain insights into the origin of vertebrate galectins. Six galectin genes are found in the genome of *P. maximus*, three on chromosome 3, two on chromosome 9, and one on chromosome 15 (**Figure 1**). Two neighboring galectins on chromosome 3, galectin-4-like (LOC117323441) and LOC117323440, have a typical bivalve galectin structure of four tandem-repeat CRDs. This particular type of galectin has previously been studied in the oyster *Crassostrea virginica*, which also has two of these proteins, CvGal1 and CvGal2 (33). Synteny, gene structure and amino acid sequence show a close relationship of these galectins between oyster and scallop. In the oyster, CvGal1 and 2 are produced by hemocytes, the phagocytes of the bivalve (34). Galectins bind exogenous glycans, for example, from pathogens, as well as endogenous glycans on the surface of hemocytes *via* their different CRDs (33). Thus, they can function as phagocytosis-mediating opsonins.

If we go evolutionary one step further toward vertebrates and consider Echinodermata as primeval deuterostomes, we found only one galectin locus on one chromosome. The sea urchin *Lytechinus variegatus* is a good model organism to assess genomic galectin structure, as it has now been completely annotated at the chromosomal level. *L. variegatus* has a classic tandem-repeat galectin with two CRDs, galectin-8-like (LOC121418813). The homologous galectin-8 (LOC576472) of the purple sea urchin *Strongylocentrotus purpuratus* could be detected in high amounts in coelomic fluid (35) and is expressed in coelomocytes, the phagocytes of Echinodermata (36). This suggests that in sea urchins, a major function of galectins is also pathogen defense. The sea cucumber *Apostichopus japonicus* has two galectins arranged in tandem in the genome. Again, these are tandem-repeat galectins with two (QCW05467, galectin-8) or three CRDs [PIK56913, AjGal1 (37)]. AjGal1 is mainly expressed in coelomocytes, binds microorganisms and has antimicrobial activity (37).

The tunicates, as the primitive chordates and closest living relatives of vertebrates, are not a good model to study the phylogenetic evolution of galectins because they are among the fastest evolving metazoans and have poor synteny conservation (38). However, *Ciona intestinalis* has three tandem-repeat galectins with two CRDs each on chromosomes 4 and 6. The galectin-6 and galectin-6-like genes are arranged in tandem on chromosome 6 and show very high sequence identity. Galectin-9 (CiLgals-a) and galectin-6 (CiLgals-b) are expressed in hemocytes of the pharynx of *C. intestinalis*. This expression is induced by inflammatory processes (39).

To date, nothing is known about the galectins of Cyclostomata on a functional level. In the genome of the sea lamprey *Petromyzon marinus*, seven galectins or galectin-related genes are found, one each on chromosomes 17, 29, and 31 and two each on chromosomes 33 and 53. Only two, galectin-8 and galectin-8-like (LOC116948446), are classical tandem-repeat galectins. In addition, a galectin-3-like (LOC116953901) and two galectin-related genes (LOC116943127 and LOC116953921) were detected with only one CRD. The uncharacterized protein LOC116947959 on chromosome 31 has four potential CRDs.

### 3.3 Exon−intron structure of bilaterian galectins

For the classification of bilaterian galectins, it is instructive to look at exon−intron organization. In Gnathostomata, it is known that each individual CRD is encoded by three exons. In tandem-repeat-type galectins, the large middle exon of the first CRD comprises the codons encoding the amino acids of β-strands S3-F4 (**Figure 1A**). In the second CRD, the middle exon is smaller and encodes S3-F3 (18). Most Gnathostomata galectins that have only one CDR correspond in their exon−intron structure to the second CRD of tandem-repeat galectins. This is the case for LGALS1, LGALS1B, LGALS2, LGALS3, Grifin, and the galectin-related genes LGALSL and LGALSL2. Only the mono-CRD galectins located in the neighborhood of LGALS4, namely, LGALS7, LGALS10, LGALS13, LGALS14, and LGALS16, correspond from the exon−intron structure to the first CRD of the tandem repeat galectins (**Figure 1B**).

In Scallop, all galectins have an exon−intron structure with a large S3-F4 coding exon (**Figure 1B**). A short S3-F3 coding exon does not occur. Exceptions are galectins with four tandem-repeat CRDs. Among these, the first CRD is encoded by only two exons. A small exon comprises the S1 and F2 β-strands, and a larger exon comprises all the others, namely, S3-F1.

Typical for deuterostomes is the appearance of the exon−intron structure characteristic of Gnathostomata galectins with a longer S3-F4 exon in the N-terminal and a shorter S3-F3 exon in the C-terminal CRD. This structure is found in tandem-repeat galectins of echinoderms, Tunicata and Cyclostomata (**Figure 1B**). Additionally, LOC116947959 of *P. marinus* encoding four CRDs has both S3-F4 (CRD1 and 3) and S3-F3 exons (CRD2 and 4). Exceptions are LGALS6 and LGALS6-like of *C. intestinalis*. Both tandem CRDs of these galectin genes have only the longer S3-F4 exon. In Cyclostomata, for the first time, in addition to the tandem-repeat type, galectins or galectin-related genes with a mono-CRD emerge. All three mono-CRD galectins of *P. marinus* have the short S3-F3 exon.

It should be mentioned that the evolutionarily more ancient Porifera, which form a sister group to Eumetazoa, also possess galectins. Exemplary is the genomically sequenced and annotated sponge *Amphimedon queenslandica*, which has been used as a model organism to study the evolution of metazoa (40). *A. queenslandica* has three galectin genes, LOC105313191, LOC109582911, and LOC105315566. All three galectins have only one CRD and are encoded by only one exon. Interestingly, all three have a signal peptide sequence (Sec/SPI) (**Supplementary Table 2**). It is typical for deuterostomes and for bivalve galectins that they do not have such a classical secretion signal. If bilateria galectins are secreted, they are transported *via* an unconventional pathway (41).

### 3.4 Amino acid sequence-based phylogenetic analysis

**Figure 2:**
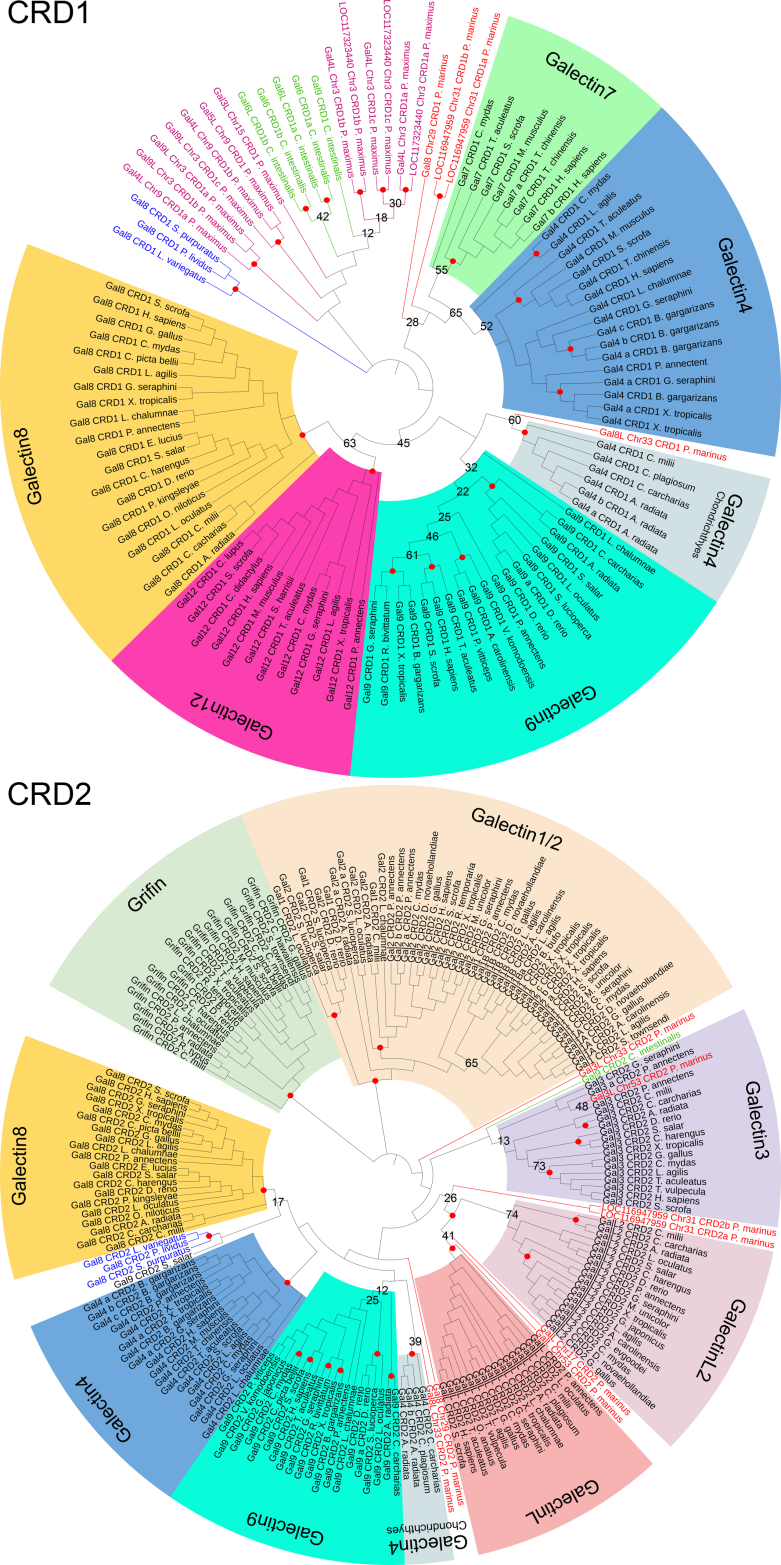
Maximum likelihood phylogenetic tree of the galectin family with 1000 bootstrap (BT) replicates. The dataset includes 107 CRD1 and 193 CRD2 amino acid sequences of 186 Gnatostomata galectins as well as the galectins of *P. marinus, C. intestinalis*, three Echinodermata species, and *P. maximus*. The individual galectin family members of the Gnatostomata are indicated by different colors. Names and branches of galectines of *P. marinus* are highlighted in red, those of *C. intestinales* in green, of the Echinodermata in blue, and of *P. maximus* in magenta. Nodes with BT values >75% are indicated by red circles. Those with lower BT values are labeled with the respective values.

To estimate the ancestral relationship between the scallop, Echinodermata, Tunicata and Cyclostomata galectins and members of the Gnathosotomata galectin family, we start with a traditional sequence alignment-based phylogenetic analysis. For this, the amino acid sequences of CRD1 and CRD2 were separately subjected to maximum likelihood analysis (**Figure 2**). As described in earlier studies (18), these analyses clearly show clustering of the individual Gnathostomata galectin groups. However, for some galectins this is only weakly supported by bootstrap (BT) analyses. This is particularly evident for galectins-3, -4 and -9, while galectin-8 and grifin, for example, show strong sequence similarity from cartilaginous fish to mammals. Looking at the individual alignments of the CRDs of galectin-3, -4 and -9, a distinct deviance in the flexible loop regions connecting the β-sheets S3 and S4 as well as S4 and S5 is particularly evident here. It is known that the amino acid residues in these loops are important for the oligosaccharide binding specificity of galectins (42). Therefore, it could be speculated that binding specificities of galectins-3, -4, and -9 are different in the Gnathostomata classes. In contrast, the high sequence homology in galectin-8 let suggest that its oligosaccharide binding specificity is highly conserved across gnathostomes.

With respect to Cyclostomata galectins, it appears that both CRD1 and CRD2 of galectin-8-like from *P. marinus* cluster to that of galectin-4 of cartilaginous fish, albeit with low BT support of 60% and 39%, respectively. In contrast, the CRD1 of lamprey galectin-8 clusters to galectin-4 of bony vertebrates, as do the two CRD1s of LOC116947959. Again, this clustering is only very weakly supported by the BT test. The CRD of galectin-3-like on chromosome 53 of *P. marinus* clusters into the galectin-3 group of Gnathostomata. The most remarkable result of this phylogenetic analysis is that both galectin-related protein-like proteins of the sea lamprey cluster with a high BT support of 99% to the galectin-related protein and galectin-related protein 2 of the jawed vertebrates (**Supplementary Figure 1**). This could be an indication that these previously understudied members of the galectin family evolved very early in the evolution of vertebrates, even before the gnathostome-cyclostome split.

However, the sequence-based phylogenetic analysis does not allow valid statements concerning a possible evolutionary ancestry of the vertebrate galectines from the more basal deuterostomes and scallops. The CRD1 of Echinodermata, *C. intestinalis* and the scallop *P. maximus* outgroup to the vertebrate galectins without a clear ancestral relationship among them. Furthermore, the CRD2 of the galectin-8 proteins of the Echinodermata cluster only very weakly to the galectin-8 proteins of the Gnathostomata.

Due to the well-known limitations of sequence-based analyses (43), we characterize the path of galectin genes through evolution by considering synteny.

### 3.5 Syntenic analyses

To investigate the origin of galectins in higher vertebrates, we looked at the chromosomal distribution of genes conserved in Gnathostomata colocalized with each galectin in the more primitive bilaterian species. Interestingly, almost all of the genes colocalized with the galectins LGALS3, LGALS8, LGALS12, LGALSL, and LGALSL2 in Gnathostomata were found on chromosome 3 in *P. maximus* (**Supplementary Figure 2A**). Three galectin genes are located on this chromosome in the scallop. The Gnathostomata genes colocalized with Griffin and LGALS1 and LGALS2 are on chromosome 1 in *P. maximus*, and those of LGALS9 are on chromosome 8. There is no galectin gene on any of these *P. maximus* chromosomes. The only galectin of the echinoderm *L. variegatus* is located on chromosome 7, and all galectin synteny genes of *P. maximus* chromosome 3 are also found on this chromosome (**Supplementary Figure 2B**). This suggests that this *L. variegatus* chromosome 7 has its origin in chromosome 3 of *P. maximus* and that the galectin-8 of the sea urchin can be traced back to one of the three bivalve galectins located there. Both the scallop and the sea urchin have 19 chromosomes. Apparently, the other *P. maximus* galectins on chromosomes 9 and 15 were not inherited to the deuterosomes. Synteny analyses in the ancestral chordates, the tunicates, are unfortunately not useful for our analyses because these organisms underwent extensive genomic rearrangements compared to the other chordate subphyla.

**Figure 3:**
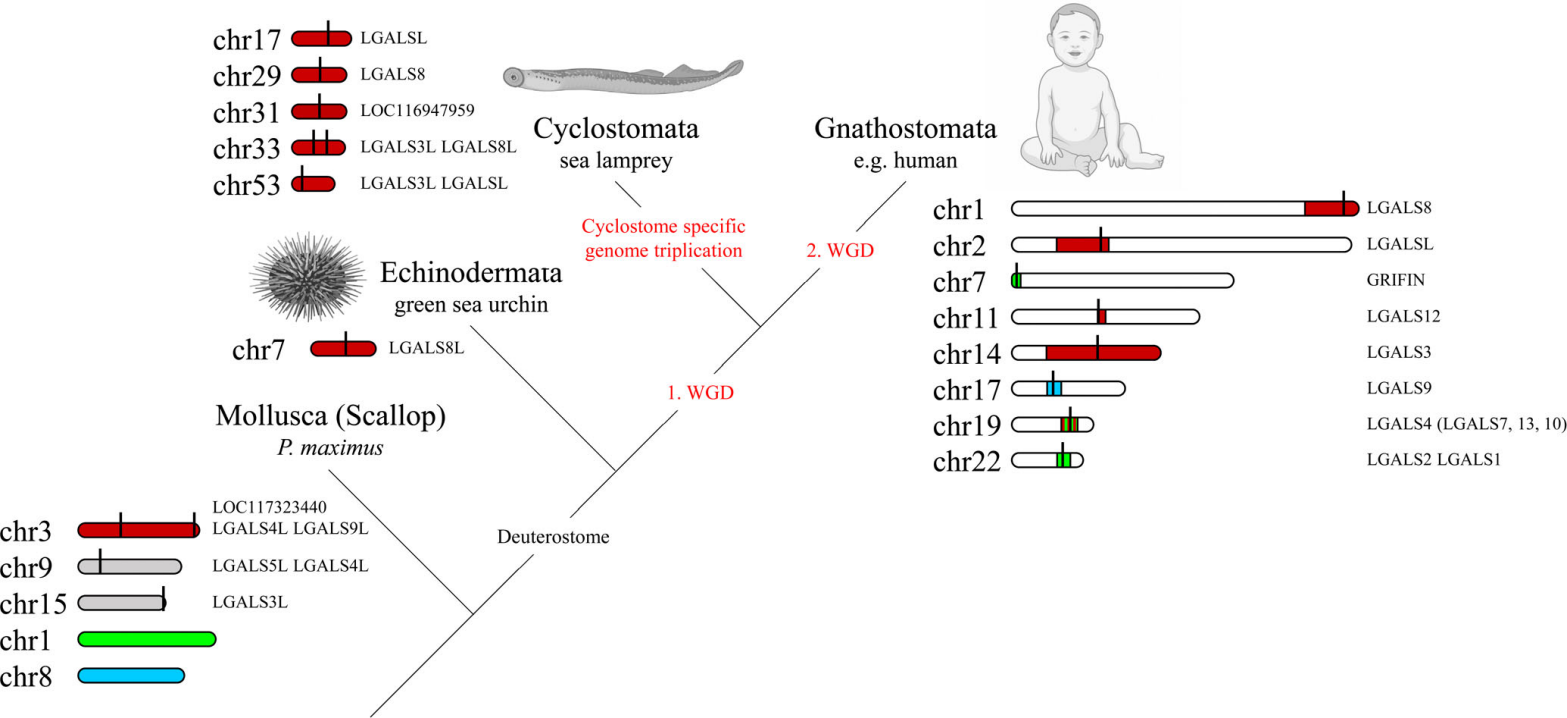
Macrosynteny Conservation of Galectin Gene-Containing Chromosomes or Chromosomal Regions of Selected Bilaterian Species. The relevant chromosomes of the scallop *P. maximus* are colored to show the homology with the chromosomes or chromosomal regions of the green sea urchin *L. variegatus*, the sea lamprey *P. marinus* and humans (stripes indicate homologies to multiple scallop chromosomes). The exact localization of the respective galectins on the chromosomes is marked with a black line. The visualization of the homologies of the chromosomal regions was adapted from Nakatani et al. (21). Created with BioRender.com.

In vertebrates, the analysis of galectine genes in the genome becomes more complex due to ancient polyploidization events. During the evolution from invertebrate chordates to gnathostomes, two rounds of WGDs occurred, which tremendously increased the complexity of the genomes. It is suggested that the gnathostome-cyclostome split occurred most likely soon after the 1^st^ WGD, followed by cyclostome-specific genome triplication (21). Interestingly, the genomes of modern lampreys seem to indicate remarkably low rates of interchromosomal rearrangement following hexaploidization (21). The sea lamprey *P. marinus* has a total of seven galectins on five chromosomes (**Supplementary Figure 2C**). Since the colocalized genes known from the scallop and sea urchin are also found on these chromosomes, it can be concluded that these genomic regions can be referred back to chromosomes 3 of *P. maximus* and 7 of *L. variegatus*, respectively (**Figure 3**). This suggests that the lamprey galectins represent paralogs of a protovertebrate galectin that is likely related to the Echinodermata galectin. The paralogs probably arose from both polyploidy events and small-scale duplication (44).

Among the gnathostomes, there are nine galectins or galectin-related proteins widely distributed across the different classes, which we have examined in more detail. These are the tandem-repeat galectins LGALS4, 8, 9 and 12, the prototypical galectins LGALS1/2 and Grifin, LGALS3 as the only representative of the chimera and the galectin-related proteins LGALSL and LGALSL2 (also referred to as LGALSLA). We further consider LGALS1 and LGALS2 as one locus, since they emerged in tandem late from Dipnotetrapodomorpha onward. In addition, a number of other galectins have arisen in the individual Gnathostomata classes by small-scale duplication. One example is LGALS7, which arose in the Amniota probably by tandem duplication of the N-terminal CRD of LGALS4.

Since Gnatosthomta have undergone two tetraploidization events during their evolution, theoretically four ohnologues should have arisen from the single ancestral galectin locus. In addition, after each of the two genome duplications, extensive chromosome rearrangements (fusions, fissions and translocations) have occurred, which complicates the identification of the ohnologues or can also lead to the loss of ohnologues. Nakatani et al. (21) were able to assign chromosomal regions of modern gnathostomes, such as the 22 chromosomes of humans, to the proto-invertebrate chromosomes they reconstructed, and these in turn to homologies to the chromosomes of the scallop genome. In this study, however, reference is made not to the *P. maximus* but to the *Chlamys farreri* genome. Both scallops belong to the order Pectinida, but the *C. farreri* genome is not available at the chromosome level in the NCBI database. However, both scallop genomes are highly homologous and readily comparable. We mapped the location of the individual human galectins against the chromosomal regions found by Nakatani and colleagues that correspond to proto-invertebrate and scallop chromosomes, respectively. Consistent with our gene-level synteny studies, we recognized that LGALS8 (Chr. 1), LGALSL (Chr. 2), LGALS12 (Chr. 11), as well as LGALS3 (Chr. 14) are located in regions corresponding to *P. maximus* and *C. farreri* chromosome 3, respectively (**Figure 3**). Human LGALS4 on chromosome 19 is located in a region that has homologies to chromosome 3 as well as to chromosomes 1 and 6 of *P. maximus* and *C. farreri*, respectively. Grifin (Chr. 7) and LGALS1 and 2 (Chr. 22) of humans are also located in regions with homologies to chromosomes 1 and 6 of *P. maximus* and *C. farreri*, respectively. LGALS9 of humans, in turn, is located on chromosome 17 in a region with macrosynteny to chromosome 8 of *P. maximus* and 9 of *C. farreri*. Moreover, most of the nearest co-localized synteny genes of LGALS9 are located on chromosome 38 of *P. marinus* [LOC116950292 (wsb1), LOC116950294 (ksr1), LOC116950297 (nos2), LYRM9] or chromosome 2 of *L. variegatus* [LOC121408157 (nf1), LOC121408172 (wsb1), LOC121408977 (ksr1), LOC121409155 (nos2), LOC121409211 (lyrm9)]. No galectin gene was found on any of these chromosomes. It should be noted that in most Gnathostomata classes, LGALS8 is associated with LGALSL, and LGALSL2 is associated with LGALS3 on one chromosomal segment. During mammalian evolution lineage-specific rearrangements, chromosome fission and fusion are known to have occurred. Thus, in this Gnathostomata class, LGALS8 and LGALSL are localized on different chromosomes, while LGALSL2 has been lost. In this respect, however, mammalian genomes are an exception.

### 3.6 The sialic acid binding domain of Galectin-8 is typical for Deuterostomia

**Figure 4:**
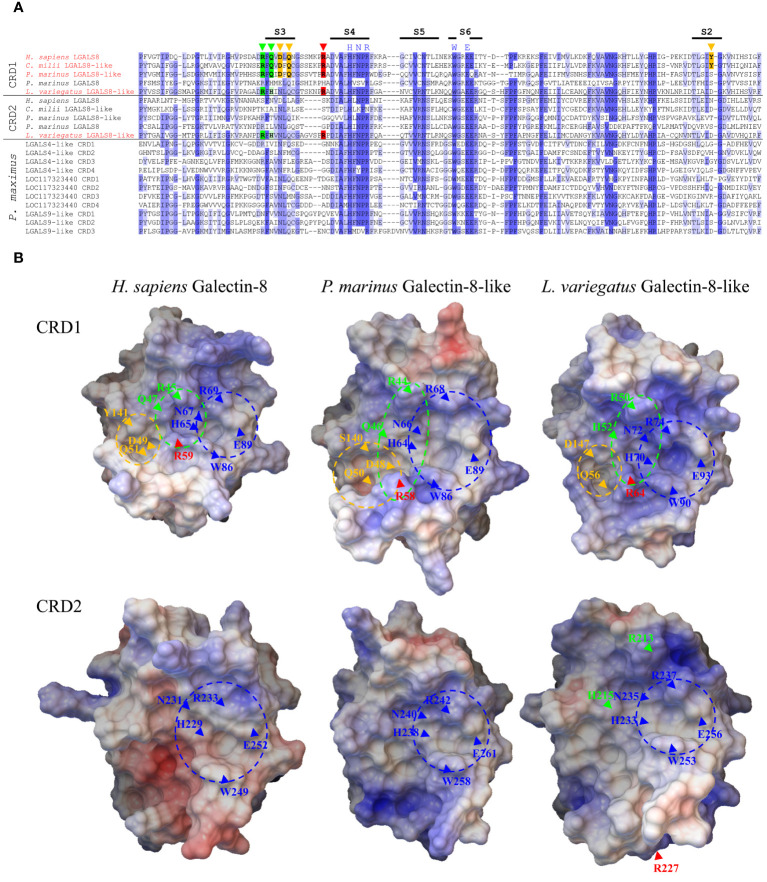
Comparison of the Primary and Tertiary Structure of Human Galectin-8 with Selected Deuterostomata and Scallop Galectins. **(A)** Sequence alignment of CRDs of galectin-8 and 8-like proteins from *H sapiens*, *C milli*, *P. marinus* and *L. variegatus* as well as the *P. maximus* galectins localized on chromosome 3. The intensity of the blue shades with which the amino acids are labeled illustrates the degree of conservation. Amino acids relevant for the binding of sulfated and sialylated oligosaccharides and for the binding of extended carbohydrates are marked in green and yellow, respectively. The arginine of the long S3-S4 loop, which is critical for the sialic acid interaction, is highlighted in red. The positions of β-strands S2-S6 involved in carbohydrate ligand binding are indicated as black bars above the alignment. **(B)** 3D models of the CRDs of human galectin-8 and the galectin-8-like proteins of *P. marinus* and *L. variegatus*. The electrostatic potential of the protein surface is indicated by red (negative) and blue (positive) colors. Regions corresponding to the known subsites of the ligand binding grooves of human galectin-8 are indicated by dashed circles. The positions of amino acids relevant for binding are indicated by arrows. Subsite and amino acids relevant for lactose recognition marked in dark blue, for sialylated and sulfated oligosaccharides in green (red, critical arginine for strong affinity to sialic acid of human galectin-8 CRD1), and those for extended oligosaccharides in yellow.

In terms of its amino acid sequence, tandem-repeat galectin-8 is one of the most conserved galectins within gnathostomes. It is essential for the vertebrate lineage and is found in all classes of jawed vertebrates. LGALS8 plays a critical role in intracellular pathogen defense as well as autophagy (9) and thus shows ubiquitinated tissue expression. This suggests that LGALS8 also appears to be one of the most primordial galectins, if not the true “proto-type” galectin of the Deuterostomata. The N-terminal CRD (CRD1) of LGALS8 is also unique with respect to its glycan-binding specificity. It has a strong affinity for sialylated glycans, whereas in the case of most other galectins, sialylation counteracts glycan-galectin binding (45). This is due to a positively charged subsite of LGALS8 CRD1. Relevant for this sialic acid binding in human LGALS8 is Arg45 and Gln47 in the S3-sheet as well as Arg59 in a long loop between the S3 and S4-sheets. Here, Arg59 is the crucial amino acid for the binding of the acidic sugar (5, 46). To determine whether this binding motif is also present in the tandem-repeat galectins of Cyclostomata and Echinodermata and in the galectins localized to chromosome 3 of *P. maximus*, we compared the CRDs of *P. marinus* LGALS8-like (LOC116948446) and LGALS8, *L. variegatus* LGALS8-like, and *P. maximus* LGALS4-like, LOC117323440, and LGALS9-like with those of human LGALS8 (**Figure 4A**). To include a rather primordial Gnathostomata LGALS8 in the comparison, we used LOC121848525 (LGALS8-like) from *Callorhinchus milii*, a representative of the Chondrichthyes subclass Holocephali (chimaera). As expected, *C. milii* LGALS8-like shows very high sequence homology to the human protein. The two crucial arginines as well as glutamine are found at the corresponding positions. In addition, the corresponding amino acids to Tyr141 in the S2-strand as well as Asp49 and Gln51 in the S3-strand, which are crucial for the binding of longer oligosaccharides in human LGALS8 (5), are also found in *C. milii*. The tyrosine in the S2-strand is particularly important because it establishes van der Waals interactions with the galactose ring. *P. marinus* LGALS8-like also possesses the arginine residues relevant for sialic acid binding as well as the corresponding glutamine. The amino acids of the S3-strand, which are necessary for the formation of the subsite for the binding of longer oligosaccharides, are also present. However, the crucial tyrosine is missing in the S2-strand. The LGALS8-like protein of *L. variegatus* also has these essential arginine residues for sialic acid binding. However, the glutamine in the S3 strand, which is also known to be involved in sialic acid binding of human galectin-8, is replaced by histidine. Interestingly, in this galectin, the relevant amino acid residues are also present in CRD2. Compared to the other studied deuterostomata LGALS8 proteins, only the CRD2 of *L. variegatus* possesses a long loop between S3 and S4 that is characteristic only of the N-terminal CRD of LGALS8. Since the arginine in the long loop is particularly important for the interaction with acidic carbohydrates, it could be speculated that this galectin can form a sialic acid binding subsite in both CRD1 and CRD2. The relevant amino acids for the formation of the subsite for binding longer oligosaccharides are not present in the *L. variegatus* galectin. In none of the *P. maximus* galectins are the conserved amino acids necessary for sialic acid binding or binding of longer oligosaccharides found. This galectin property does not yet appear to be present in this protostomes.

Sialic acids are found prominently expressed in deuterostomes and almost absent in the protostome lineage (47). In accordance with this, sialyltransferases, the enzymes that add sialic acid resides to nascent glycans, are only present as a large gene family in deuterostomes (48). It is speculated that the sialylation machinery evolved in the last common ancestor of metazoa before the separation of protostomes and deuterostomes. Subsequently, it appears to have been largely lost in protostomes (49). The wide expansion of this gene family in deuterostomes seems to indicate the importance of sialoglycoconjugates in this lineage. This could also explain the evolution of sialic acid-binding galectins in deuterostomes, most likely as part of the self-recognition machinery of the initially still primitive immune system. This could be of particular relevance since Siglecs (sialic acis-binding immunoglobulin-type lectins), as the most prominent group of sialic acid-binding proteins, probably first evolved in Gnatostomata (50).

Considering the predicted 3D structure of the CRDs of the galectin-8 proteins from humans, *P. marinus* and *L. variegatus*, a positively charged carbohydrate binding groove can be identified in the N-terminal CRDs (**Figure 4B**). Additionally, clearly visible are three subsite pockets, which mediate the binding of lactose (blue site), sialylated and sulfated oligosaccharides (green site) and extended carbohydrates of longer oligosaccharides (yellow site) in human galectin-8 (5). Furthermore, the arginine residue of the long S3-S4 loop (red arrow), which is critical for sialic acid binding, is found at the N-terminal CRDs of all three galectins in close proximity to the highly conserved tryptophan residue in the S6-strand, whose aromatic ring is important in stacking interactions with galactose. Interestingly, the 3D model of the C-terminal CRD of the *L. variegatus* galectin-8-like protein shows that arginine R227 of the long S3-S4 loop (**Figure 4B**, red arrow) is not located near the tryptophan of the S6-strand. This indicates that the sialic acid binding property of this CRD2, which was assumed based on the amino acid sequence, is obviously a misinterpretation.

### 3.7 Hypothetical model of the evolution of gnathostomata galectin cosmos

**Figure 5:**
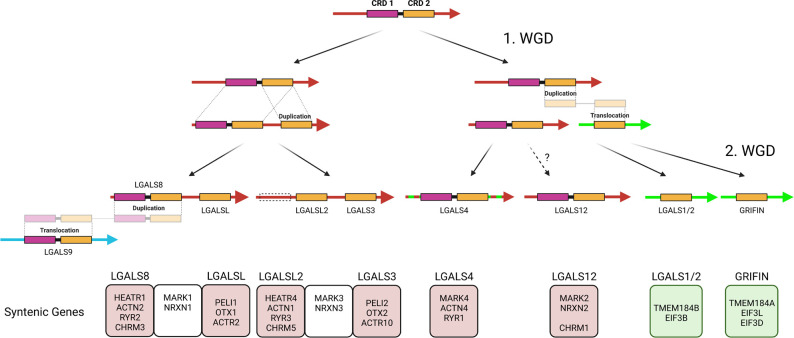
Schematic Representation of the Hypothesis of Gnathostomata Galectin Genesis from the Protovertebrate Ancestor. The color of the arrows indicates the homology of chromosomal regions with *P. maximus* chromosomes 3 (red), 1 (green) and 8 (blue). Syntenic genes that, based on their ohnology and position, allow predictions about the origin of the respective galectins during the two rounds of WGD as well as their evolutionary relationship are presented in boxes below. Created with BioRender.com.

Based on the assumption that the proto-invertebrate ancestor, similar to Echinodermata, possessed only one galectin locus in the genome, we have attempted to elicit the evolutionary complexity of the galectin family of modern Gnathostomata *via* synteny comparisons (**Figure 5**). The tandem-repeat galectin typical of Deuterostomia, with an N-terminal CRD characterized by a large S3-F4 exon and a C-terminal CRD possessing a smaller S3-F3 exon, should also have been present in the proto-vertebrate ancestor. During the 1^st^ round of WGD, this tandem-repeat galectin was duplicated together with the whole chromosome. Thereafter, the proto-cyclosostomes split off. Chromosomal rearrangements and fusions subsequently occurred in the proto-gnastostomata lineage (21). The C-terminal domain of one of the duplicated tandem-repeat galectins was duplicated in tandem. Furthermore, another C-terminal CRD was duplicated and translocated to a chromosomal region that has synteny to chromosome 1 of *P. maximus*. The chromosomal region in which human LGALS4 is located appears to have synteny with both chromosome 3 and chromosome 1 of *P. maximus*. This could be due to chromosome fusions that may have occurred after the 1^st^ round of WGD. If so, this would explain a subsequent translocation of a duplicated CRD into chromosomal regions corresponding to scallop chromosome 1. At the end of chromosomal remodeling after the 1^st^ WGD, a locus with a tandem-repeat galectin together with a prototype galectin consisting of only the C-terminal CRD should be present. Furthermore, a locus with only a single tandem-repeat galectin and another locus with a prototype galectin on a different chromosome should also have existed. In the subsequent 2^nd^ WGD round, these three loci were again duplicated. Two double-galectin loci consisting of LGALS8 and LGALSL or LGALSL2 and LGALS3, which have the same origin, can be detected in most Gnathostomata classes *via* synteny. This fact has been overlooked in previous studies because LGALSL2 has been lost in Mammalia and, thus, has never been the focus of galectin research. In LGALSL2, the N-terminal CRD has been lost. A common origin can also be identified for the LGALS1/LGALS2 and Grifin loci *via* synteny. However, these genes are located in a genomic region unrelated to that of the proto-invertebrate, Echinodermata and bivalve galectin. This aspect of the close evolutionary relationship between LGALS1/2 and Grifin has also hardly been considered. The lens-specifc Grifin has lost the crucial galactose binding motif in mammals. This loss of galectin-typical carbohydrate-binding property makes Grifin of little interest for galectin research. However, Caballero et al., 2018 inferred a relationship between Grifin and LGALS1, LGALS2 and LGALS3 in rats by analyzing amino acid sequence similarities (51). In contrast, the syntenic genes of LGALS4 and LGALS12 clearly indicate a close relationship with the two tandem-repeat galectins of the double loci. At first glance, the origin of LGALS12 is problematic because this galectin is only found in the Dipnotetrapodomorpha, i.e., only from the lungfish onward. However, if we look at the syntenic genes of this galectin in the more primitive, chromosome-level annotated Gnathostomata genomes, we observed that they are often localized on microchromosomes. Microchromosomes have the characteristic that they combine a very high gene density with a high GC content and repeat richness. These properties pose a major problem in whole genome sequencing. Only recently, a new genome analysis conducted by the “Vertebrate Genomes Project” (https://vertebrategenomesproject.org/), which uses high-resolution genome sequencing methods and a more elaborate analysis pipeline, found that these newer methods can be used to identify a tremendous number of genes and genome regions that were missed in previous assemblies (52). For example, it was shown that eight new microchromosomes and 400 previously missed genes could be identified in zebra finch compared to old assemblies. Of particular note, these missing genes account for up to 50% of the genes in the microchromosomes. The reason for this is that GC-rich and repetitive regions are a major problem in classical genome sequencing since they initiate secondary structures. Thus, previous assemblies often miss or misrepresent genes and sometimes even whole chromosomes. Therefore, it could be assumed that LGALS12 might exist in Chondrichthyes or, for instance, *Lepisosteus oculatus* but could not yet be identified. Furthermore, there is also the possibility that LGALS12 has actually been lost in the course of the individual evolution of these animal classes.

LGALS9 is the last of the nine major galectins of Gnathosomata. This galectin is located in a chromosomal region corresponding to chromosome 8 of *P. maximus* and differs from all other nine galectins with respect to its localization. It is very likely that it arose by duplication and subsequent translocation of one of the tandem-repeat galectins after the completed 2^nd^ WGD. Based on the sequence similarity, it can be assumed that LGALS8 represents the donor gene. In most previous studies, LGALS9, based on its structure, is classified as one of the four ohnologues of a proto-vertebrate tandem-repeat galectin, together with LGALS4, 8, and 12. Based on the more recent macrosynteny data, we conclude that this is not the case.

## 4 Conclusion

Galectins have evolved within Bilateria from proteins originally functioning as opsonins to a large family of regulatory factors of diverse physiological processes. In Molluska and in the invertebrate Deuterostomata, the main function of galectins seems to be the initiation of phagocytosis of pathogens. For this mechanism of innate immune defense, galectins bind both pathogens and primitive immune cells. It can be assumed that in vertebrates, together with the formation of a more complex immune system and the development of the adaptive immune response with very different cell types, the immunoregulatory roles of galectins have become more important. We were able to infer how this vertebrate galectin cosmos fanned out using various approaches, particularly synteny analyses. Notably, we also included previously neglected galectins, such as the galectin-related protein LGALSL2. This galectin deserves further analysis in the future, including functional analysis, as it may have broader relevance in all other jawed vertebrates except Mammalia. Furthermore, we hope that the results of our study will direct the attention of future research not only to the classical galectins, but also to the galectin-related proteins, which have so far been rather neglected. These proteins seem to have evolved early in vertebrate evolution and therefore probably belong to the basic set of vertebrate galectins. However, little is known about their physiological function. Moreover, a closer look at the galectins of Cyclostomata as a sister group of gnathostomes could provide interesting insights into the function of the immune system. Cyclostomata, similar to Gnathostomata, have convergently evolved an adaptive immune system (53). This is strikingly different from that of jawed vertebrates. How galectins interact here could reveal interesting new aspects of the regulatory function of this important protein family.

## Data availability statement

The original contributions presented in the study are included in the article/**Supplementary Material**. Further inquiries can be directed to the corresponding author.

## Author contributions

JG and SG conceived the study. JG performed the analyses and wrote the original draft, JG and SG reviewed and edited the manuscript. All authors contributed to the article and approved the submitted version.

## Supplementary material

Supplementary Figure 1 Multiple sequence alignment of the CRD of the galectin-related protein and galectin-related protein 2 of Gnatostomata and the two galectin-related protein-like proteins of *P. marinus*. Amino acids are colored according to the Clustral color scheme.

Supplementary Figure 2 Schematic Overview of Chromosomal Localization of Galectins and Their Syntenic Genes of Scallop, Green Sea Urchin and Sea Lamprey. (A) *P. maximus* chromosome 3 (NC_047017.1), (B) *L. variegatus* chromosome 7 (NC_054746.1), and (C) *P. marinus* chromosomes 17 (NC_046085.1), 29 (NC_046097.1), 31 (NC_046099.1), 33 (NC_046101.1), and 53 (NC_046121.1). Red, galectin genes.

Supplementary Table 1 List of galectins used in the phylogenetic analyses.

Supplementary Table 2 Signal peptide prediction of galectins using SignalP-5.0 (Eukarya) (https://services.healthtech.dtu.dk/service.php?SignalP-5.0) (54).
