# Supplementary figures and images for: A brief history of galectin evolution
Source: Front Immunol. 2023 Jun 29;14:1147356. doi: 10.3389/fimmu.2023.1147356 (PMC10343441; doi:10.3389/fimmu.2023.1147356)

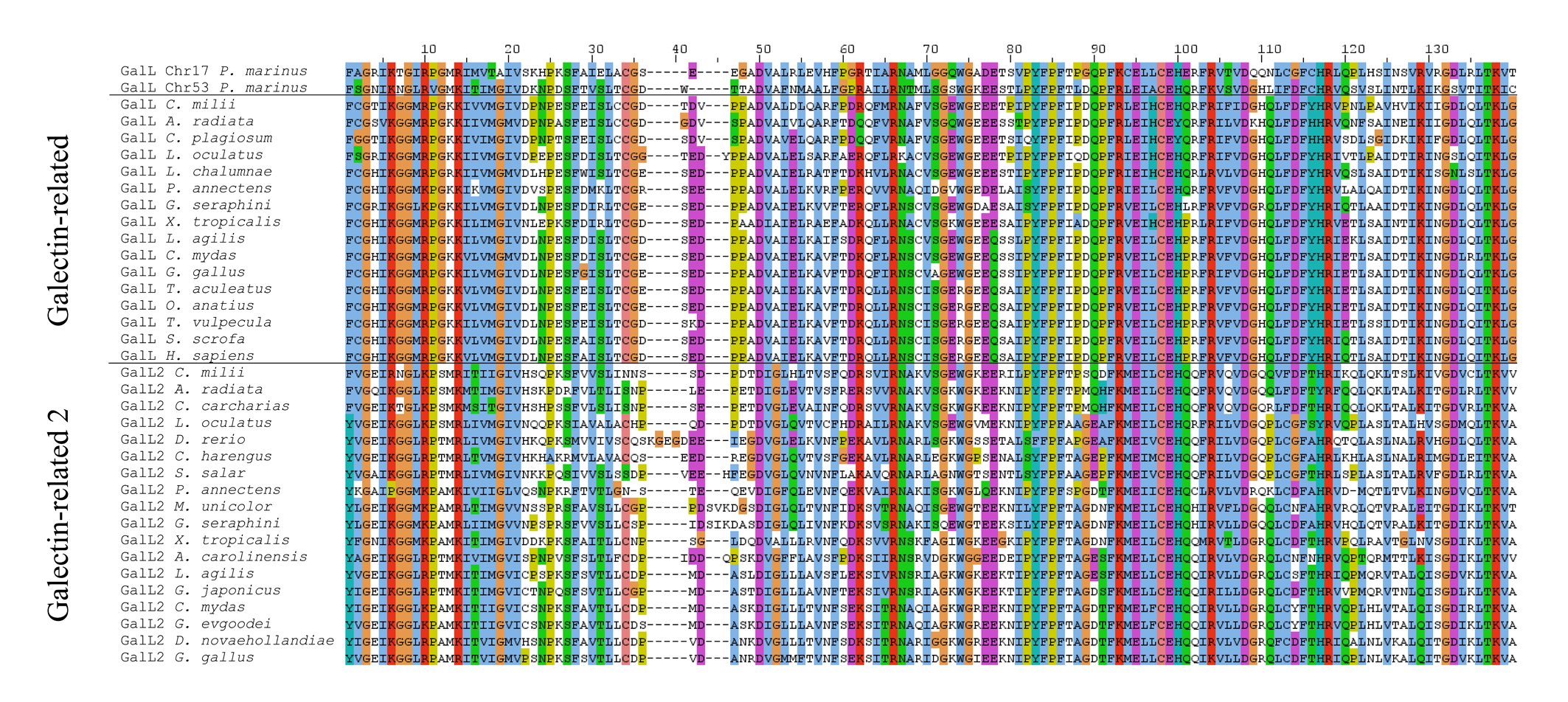

Supplement: Supplementary Figure 1 — Multiple sequence alignment of the CRD of the galectin-related protein and galectin-related protein 2 of Gnatostomata and the two galectin-related protein-like proteins of P. marinus. Amino acids are colored according to the Clustral color scheme. [file Image_1.tif]

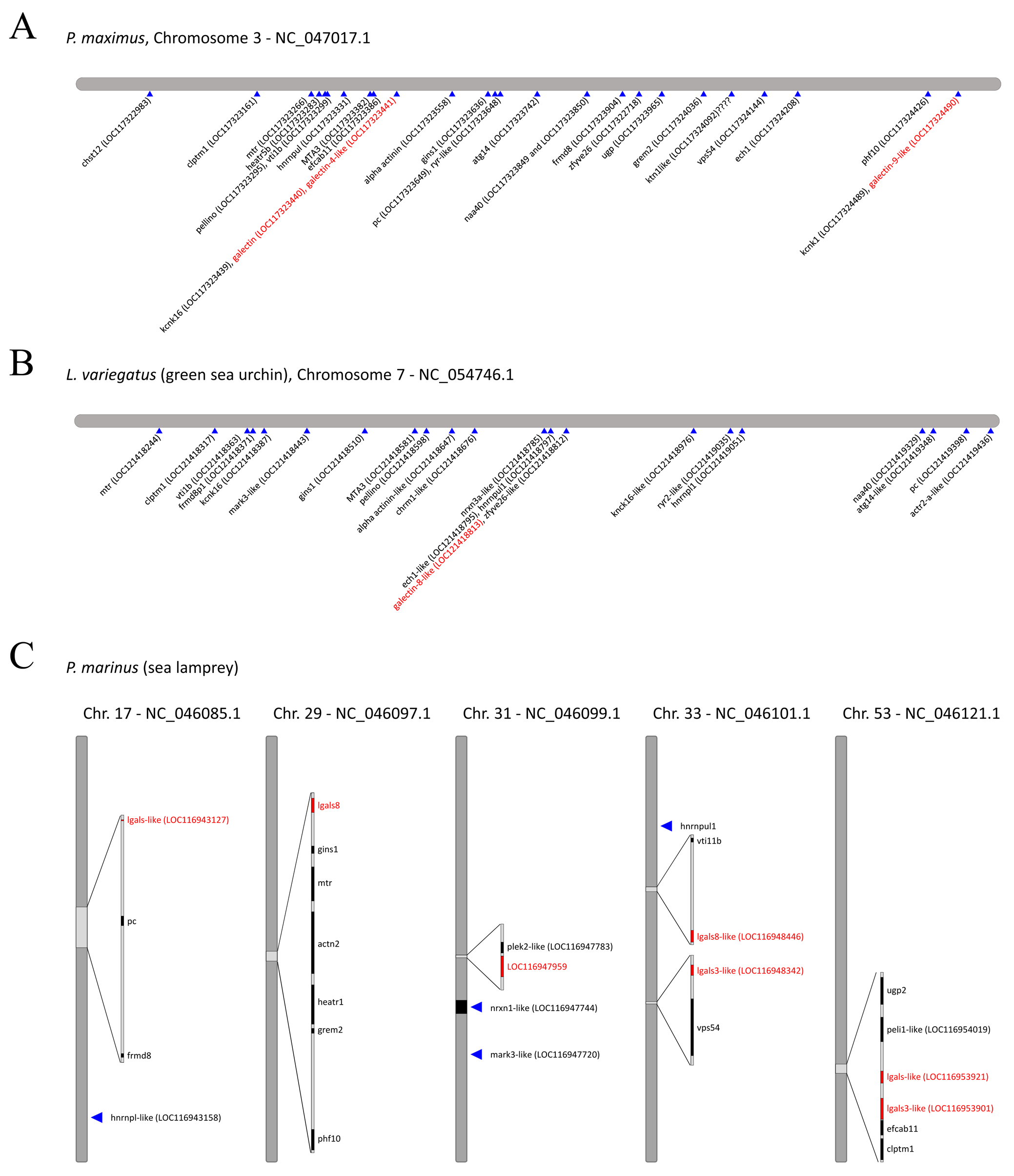

Supplement: Supplementary Figure 2 — Schematic Overview of Chromosomal Localization of Galectins and Their Syntenic Genes of Scallop, Green Sea Urchin and Sea Lamprey. (A) P. maximus chromosome 3 (NC_047017.1), (B) L. variegatus chromosome 7 (NC_054746.1), and (C) P. marinus chromosomes 17 (NC_046085.1), 29 (NC_046097.1), 31 (NC_046099.1), 33 (NC_046101.1), and 53 (NC_046121.1). Red, galectin genes. [file Image_2.tif]
